# Mechanisms of Eosinophil Degranulation

**DOI:** 10.3390/cells15131211

**Published:** 2026-07-03

**Authors:** Sarah Almas, Paige Lacy

**Affiliations:** 1 Department of Otolaryngology, Head and Neck Surgery, University of Alberta, Edmonton, AB T6G 2B7, Canada; almas1@ualberta.ca; 2 Division of Pulmonary Medicine, 559 Heritage Medical Research Centre, Department of Medicine, University of Alberta, Edmonton, AB T6G 2R3, Canada

**Keywords:** exocytosis, crystalloid granules, compound exocytosis, piecemeal degranulation, cytolysis, exosomes, extracellular traps, kinases, Rac guanosine triphosphatases, Rab guanosine triphosphatases, SNAREs, actin cytoskeleton, cytokines

## Abstract

Eosinophils are highly granulated white blood and tissue cells that play complex roles in the immune system including host protection against helminthic parasites, viruses, fungi, and bacteria. These bone marrow-derived cells cause tissue damage in a range of diseases and disorders, particularly in allergy, asthma, and chronic rhinosinusitis with nasal polyps. Eosinophils are recruited to tissues in response to chemotactic signals, and during inflammation, they release a plethora of mediators, including immunoregulatory cytokines, through multiple pathways involving degranulation, respiratory burst, lipid mediator release, exosome release, and extracellular trap formation. Degranulation from eosinophils has been implicated as a major effector mechanism in airway diseases, particularly late phase asthma responses and in nasal polyps from patients with chronic rhinosinusitis. In degranulation responses, eosinophils release numerous granule proteins by classical exocytosis, compound exocytosis, piecemeal degranulation, and cytolysis, which refers to cell lysis through membrane rupture and cell destruction. Cytolysis can lead to suicidal extracellular trap formation, which is a regulated form of cell death involving the release of extracellular DNA traps and granule proteins. Granule release from eosinophils is dependent on activation of specific and tightly regulated intracellular signaling pathways, including Rac and Rab guanosine triphosphatases, soluble NSF attachment protein (SNAP) receptors (SNAREs), Cdk5 kinase, and actin dynamics. These observations have shown selective and nonredundant roles for signaling in degranulation responses. In this review, we explore findings from the literature on the mechanisms controlling granule-derived mediator release from eosinophils.

## 1. Introduction

Eosinophils are highly granulated white blood cells that move through systemic circulation to emigrate to peripheral tissues. They arise in the bone marrow from myeloid progenitors in response to specific cytokines, including interleukins (IL)-5 (gene symbol *IL5*), IL-3 (*IL3*) and granulocyte-macrophage colony-stimulating factor (GM-CSF, *CSF2*) in humans and mice, on which the majority of research on eosinophil biology has been carried out [1,2]. IL-5 is a key regulator of eosinophilopoiesis, and it serves to promote eosinophil lineage expansion, survival, activation, and tissue accumulation. Traditionally, IL-5 was considered essential for eosinophil maturation and survival in human and mouse studies [3], but more recent literature suggests that eosinophil terminal maturation can occur in the absence of IL-5 [4]. In healthy individuals, eosinophils make up a small proportion of white blood cells (0–3%), but they become elevated in specific conditions associated with type 2 immune responses (T2). T2 immune responses involve excess production of interleukins (ILs) such as IL-4 and IL-5, which are essential for inducing the proliferation and maturation of eosinophils in the bone marrow, along with elevated chemokines that attract eosinophils to target tissues including CCL5 (RANTES, *CCL5*) and CCL11, CCL24, and CCL26 (eotaxins 1–3, *CCL11*, *CCL24*, and *CCL26*) [1]. In the case of allergic asthma, allergic rhinitis, and chronic rhinosinusitis with nasal polyps, which are predominantly T2-driven diseases, eosinophils accumulate in the mucosa and can be seen transmigrating into lumenal spaces [5]. Given the importance of IL-5 in eosinophil proliferation and differentiation, this cytokine is the target of monoclonal antibody approved for severe eosinophilic asthma, anti-IL-5 (mepolizumab and reslizumab) [6].

As effector cells in innate immunity, eosinophils play a role in preventing infections against a range of pathogens including helminthic parasites, bacteria, viruses, and fungi [7,8,9,10,11]. However, these cells appear to be redundant in their role in immunity as their absence does not result in infection by opportunistic microorganisms. Mouse models that lack eosinophils, such as PHIL, iPHIL, dblGATA1, and double knockout major basic protein (MBP-1, *Prg2*)/eosinophil peroxidase (EPX, *Epx*) mice do not exhibit major immune, health, reproductive, or functional deficiencies [12,13]. The surprising lack of effect by eosinophil depletion on the health of animals is mirrored in humans, which also appear to be largely unaffected by the loss of eosinophils from their immune system [14]. In contrast, when they are present in abundance in most T2-driven allergic diseases, they have tissue-damaging effects that are associated with worsened outcomes [15,16]. Moreover, the impact of eosinophils on tissues in disease is not only related to the numbers of these cells but is entirely dependent on their activation, leading to pro-inflammatory mediator release.

The definition of pro-inflammatory mediator release is the controlled secretion or production of substances derived from de novo synthesis or from sites of storage in secretory organelles. The secretion of these preformed mediators involves receptor-mediated degranulation, during which receptor activation at the plasma membrane triggers trafficking of membrane-bound organelles to the cell membrane for release of their contents. A diverse array of proteins and enzymes are released from eosinophil secretory organelles that have the capacity to kill extracellular pathogenic microorganisms. Eosinophils can also generate extracellular traps, known as EETs, composed of DNA, histones, nuclear, and granule proteins, which are highly effective at trapping and killing invasive microorganisms [17,18]. The protective properties of EETs are proposed to enhance the effectiveness of antimicrobial components by enriching them within a fibrous network that covers a large area around cells and reduces their exposure to host tissues.

The excessive release of eosinophil granule proteins is a common feature of many inflammatory disorders [19]. However, we are still learning the precise contribution of eosinophil degranulation to these disorders, as eosinophils also possess the ability to release many factors including a range of lipid mediators and reactive oxygen species. A surprising observation made recently is that depletion of eosinophils is insufficient to control the symptoms of eosinophilic esophagitis [20], despite evidence of widespread degranulation by cytolysis in esophageal tissues [21]. In contrast, eosinophil depletion by the application of anti-IL-5/IL-5 receptor biologics like mepolizumab, reslizumab, and benralizumab, is a highly effective strategy for several T2-driven diseases such as allergic asthma, chronic rhinosinusitis with nasal polyps, and hypereosinophilic syndrome [22,23,24]. Therefore, eosinophil degranulation is not always damaging to the host, depending on the context of illness, and we stand to gain more insight by examining the mechanisms of eosinophil degranulation in association with symptom control in diseases.

## 2. Granule Populations in Eosinophils

**Figure 1:**
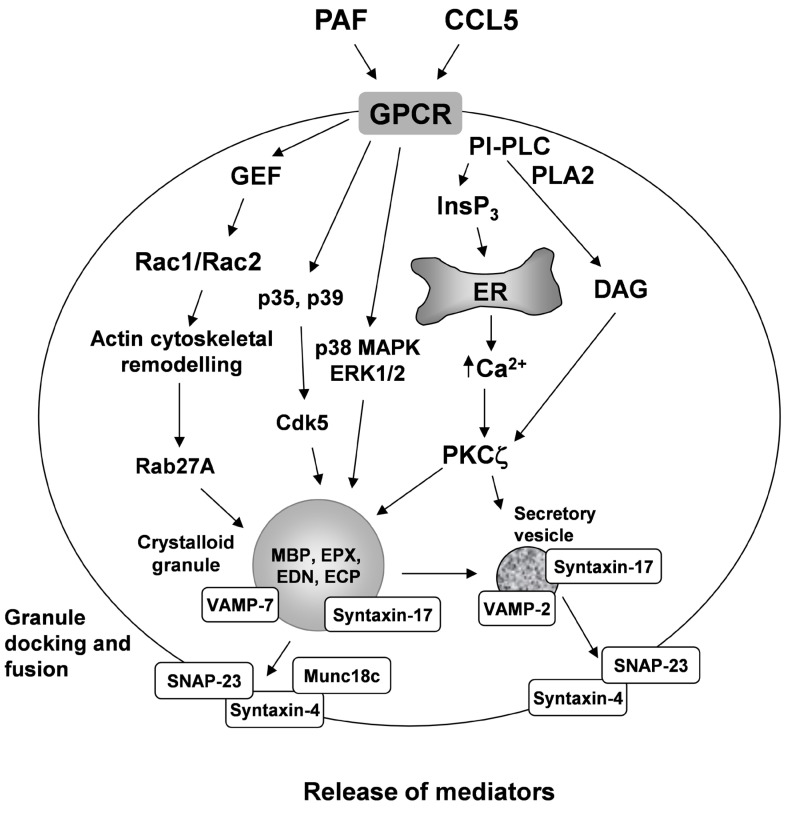
Signaling pathways involved in GPCR-dependent regulated exocytosis in eosinophils. GPCR binding by chemoattractants induces multiple overlapping intracellular pathways to regulate the selective release of eosinophil granules and vesicles. Some of these pathways are redundant in their functions, such as Rac2 which may be supported by Rac1 in Rac2-deficient eosinophils. Cdk5: cyclin-dependent kinase 5, DAG: diacylglycerol, ECP: eosinophil cationic protein, EDN: eosinophil-derived neurotoxin: EPX: eosinophil peroxidase, ER: endoplasmic reticulum, ERK1/2: extracellular signal-related kinases 1 and 2, GPCR: G-protein coupled receptor, GEF: guanine nucleotide exchange factor, InsP_3_: inositol 1,4,5-trisphoshate, MBP: major basic protein, p38 MAPK: p38 MAP kinase, PAF: platelet-activating factor, PI-PLC: phosphoinositide phospholipase C, PKC: protein kinase C, PLA2: phospholipase A2.

Eosinophils possess at least four different types of granules that appear at different stages of development. These are: (1) primary granules, which appear in developing eosinophils in the bone marrow, and may represent immature forms of specific granules, (2) small granules, that contain aryl sulfatase and acid phosphatase, (3) specific granules, also known as secondary or crystalloid granules, and (4) small secretory vesicles (Figure 1).

In eosinophils developing in the bone marrow, primary granules, also known as immature/specific granules, have been identified as large spherical bodies of 0.6–1.2 μm in diameter. These are present in early developmental stages (promyelocytes) and are occasionally found in mature cells. Primary granules are historically described to be enriched in Charcot-Leyden crystal protein (galectin-10, *CLC*) [2,25], with more recent literature suggesting that galectin-10 is also more abundantly found in the peripheral cytoplasm, separate from granular components of human eosinophils [26].

The predominant granule type in mature human eosinophils is the oval-shaped crystalloid granule measuring 0.7–1.0 μm in diameter, also known as the specific (or historically known as secondary) granule, which contains a centrally located crystalline core predominantly enriched in MBP [27,28]. This electron-dense core, as determined by transmission electron microscopy, is surrounded by a fluid matrix containing EPX, eosinophil-derived neurotoxin (EDN, *RNASE2*), eosinophil cationic protein (ECP, *RNASE3*), and other granule components [29,30]. The periphery of the granule is less electron dense. This distinctive core–matrix architecture is a defining ultrastructural feature of mature eosinophils and readily distinguishes eosinophil specific granules from that of other leukocyte populations [28].

Another major secretory organelle identified in human eosinophils is the small secretory vesicle, also known as the microgranule, which functions to shuttle crystalloid granule proteins and other newly synthesized proteins, including cytokines and chemokines, to the cell surface [19,29,30]. These are ten-fold smaller than crystalloid granules, ranging 50–100 nm in diameter, and can be rapidly moved through the cell cytoplasm to transport their protein cargo. Also known as eosinophil sombrero vesicles, these are tubular carriers that are released from eosinophils during cytolysis and persist after the disintegration of other organelles [31]. They associate with extracellular granules and externalized chromatin during EETosis and hence may play a role in the eosinophil immune responses after cell death.

Small granules have been identified in eosinophils from earlier studies that were characterized for their content of acid phosphatase and aryl sulfatase [32]. However, further ultrastructural studies have found that these small granules are actually eosinophil sombrero vesicles [33,34,35,36], a tubulovesicular transport system which originates from activated specific granules, which plays an important role in mediating the intracellular transport and secretion of granule-derived proteins during eosinophil piecemeal degranulation.

All granules are prevented from being released until receptors in the plasma membrane signal to the cytoplasm to activate their movement to the cell membrane for secretion of their contents by degranulation. This is an important regulatory mechanism as eosinophils are highly enriched in toxic tissue-damaging cationic proteins and the persistence of eosinophils in tissue underlies the pathogenesis of most chronic T2-driven inflammatory diseases.

## 3. Modes of Degranulation

Receptors expressed in the cell membrane of eosinophils can bind to numerous ligands that induce secretion. Ligands that trigger receptor-mediated secretion may be soluble or immobilized on surfaces. Soluble stimuli that activate eosinophil degranulation include platelet-activating factor (PAF), while immobilized stimuli such as immune complexes bound to surfaces can signal through Fcγ receptors in eosinophils and are highly potent [37,38]. Our studies have shown the release of cytokines from crystalloid granules and vesicles in human eosinophils of patients with allergic asthma when stimulated with PAF [39]. Other stimuli such as CCL11 and tumor necrosis factor (TNF-α) induce degranulation and compound exocytosis and increase the formation of CD63^+^ (*CD63*) large vesiculotubular carriers, or eosinophil sombrero vesicles, which fuse with granules in the process of secretion [40]. The release of preformed granule-derived mediators from eosinophils occurs by a tightly controlled receptor-coupled mechanism leading to regulated exocytosis [19].

The process of exocytosis is proposed to take place in four discrete steps [41]. The first step involves granule recruitment from the cytoplasm to the cell membrane, which is dependent on remodeling of the actin cytoskeleton and microtubules [41]. This is followed by granule tethering and docking, where the outer surface of the lipid bilayer membrane surrounding the granule contacts the inner surface of the cell membrane. Granule priming then occurs to allow granules to become fusion-competent and ensures their rapid fusion, where a reversible pore structure develops between the granule and the cell membrane. Fusion occurs when the fusion pore expands, leading to the complete fusion of the granule with the target membrane to release granule contents. When this occurs, the total surface area of the cell increases by the area of the granule membrane added to the cell membrane, and the interior of the granule membrane is exposed to the exterior surface.

Eosinophils undergo four main types of degranulation, categorized as (1) classical exocytosis or release of secretory granules and vesicles whereby individual granules fuse with the cell membrane one at a time, (2) compound exocytosis where two or more granules undergo homotypic fusion prior to release of their contents to the exterior of the cell through a single fusion pore, (3) piecemeal degranulation in which small secretory vesicles shuttle the contents of the secretory granules to the cell surface and are proposed to release their contents through exocytosis, and (4) cytolysis, or necrosis, involving the dissolution of the cell membrane and loss of intact membrane-bound secretory granules to the extracellular compartment [3,41]. The first three modes of degranulation are associated with tightly controlled receptor-mediated secretion pathways which overlap with, but are distinct from, those that govern cytolysis. Piecemeal degranulation is dependent on exocytosis of small secretory vesicles that fuse with the plasma membrane to release their intraluminal granule proteins and other de novo synthesized proteins to into the extracellular space and is the predominant degranulation mechanism observed in allergic diseases [36].

Cytolysis has also been associated with the formation of EETs in which DNA is released along with histones and granule proteins including MBP [17,42]. It is possible to induce cytolysis in human eosinophils by treating them with immobilized immune complexes together with iC3b [43], suggesting that this is a receptor-mediated mechanism and is not simply the result of spontaneous eosinophil death. In the case of exosome release, multivesicular bodies have been identified in eosinophils that fuse with the plasma membrane and release their intraluminal vesicles as exosomes into the extracellular space by exocytosis through individual fusion pores [44].

Translocation and exocytosis of granules in eosinophils require a minimum of increased intracellular Ca^2+^ and hydrolysis of guanosine triphosphate (GTP) [45,46]. There are numerous postulated target molecules for these effectors, including Ca^2+^-binding proteins such as annexins and calmodulin, as well as GTP-binding proteins such as G proteins and small monomeric guanosine triphosphatases (GTPases). Concurrently with the activation of a plethora of effector molecules that work to promote exocytosis, actin remodeling occurs with the disassembly of filamentous actin (F-actin). F-actin normally forms a barrier around the periphery of the cell and prevents granule docking and fusion. Depolymerization of the cortical F-actin polymer mesh to G-actin monomers that diffuse into the cytoplasm must occur to allow access of granules to the inner surface of the plasma membrane [47]. The process of granule translocation and exocytosis in response to receptor stimulation involves the activation and recruitment of many different signaling mechanisms, of which only a few have been identified, shown in Table 1.

## 4. Mechanisms Regulating Degranulation

### 4.1. Ca^2+^ Signaling

Increased intracellular Ca^2+^ is sufficient to induce the release of eosinophil granules, particularly if the concentration of Ca^2+^ is highly elevated using Ca^2+^ ionophores such as A23187 or ionomycin [67]. In addition, high intracellular Ca^2+^ concentrations can be achieved by permeabilizing cells with a pore-forming reagent such as streptolysin-*O*, a pore-forming toxin produced by group A streptococcus, in the presence of Ca^2+^ buffers that have elevated Ca^2+^ concentrations sufficient to promote cell activation without causing lysis [45]. The induction of granule exocytosis by Ca^2+^ is further enhanced by the addition of a nonhydrolyzable analog of GTP, known as GTPγS, underscoring the fundamental role of GTP-binding proteins in exocytosis. Moreover, patch clamp experiments using Ca^2+^ buffers and/or GTPγS loaded into cell-attaching pipettes have demonstrated that classical exocytosis can be induced in eosinophils [68,69,70]. Activation of eosinophil degranulation through the whole cell patch clamp configuration leads to a stepwise increment in membrane capacity for the whole cell, indicating that individual granules in activated cells fuse with the cell membrane in a sequential manner. Compound exocytosis has also been observed in patch clamp experiments when intracellularly applied doses of Ca^2+^ and GTPγS are elevated further, in which substantially larger steps in capacitance can be observed [71].

Under physiological conditions, eosinophils preferentially release their granule-derived mediator contents through small secretory vesicles via piecemeal degranulation over that of complete exocytosis of their crystalloid granules [36]. Many eosinophil receptors activate the increase of Ca^2+^ from extracellular and intracellular sources, including the seven transmembrane-spanning G protein-coupled receptors such as the PAF receptor (*PTAFR*) and chemokine receptors (for example, CCR3). Although Ca^2+^ is a crucial second messenger for the activation of exocytosis in eosinophils, the specific targets for Ca^2+^ in degranulation have yet to be identified.

### 4.2. Phospholipid Signaling

Numerous phospholipids are potent secretagogues for eosinophils including PAF, LysoPAF, and lysophosphatidylcholine (LPC) [38]. These mediators induce mouse and human eosinophil degranulation through a PAF receptor-dependent mechanism in addition to a PAF receptor-independent mechanism, at least in mouse eosinophils [38]. Al-though the additional receptor(s) for PAF in eosinophils remains unknown, the literature has demonstrated that PAF-induced eosinophil degranulation in humans and mice is not blocked by PAFR antagonists or PAFR knockout in mouse eosinophils [38]. Furthermore, a signaling lysophospholipid called lysophosphatidylserine (LysoPS) has been shown to induce degranulation via the purinergic P2Y10 G-protein coupled receptor more potently in eosinophils obtained from patients with severe asthma than in nonsevere asthma, suggesting that P2Y10 is involved in lysophospholipid signaling that may cross-talk with the PAF receptor [72].

A role for intracellular membrane phospholipids, particularly phosphoinositides, has been demonstrated in regulating eosinophil degranulation responses. Various types of phosphoinositides, particularly phosphatidylinositol 4,5-bisphosphate (PIP_2_), may be acted on by receptor-mediated activation of phosphatidylinositol-specific phospholipase C (PI-PLC, *PLCB1-4*) which has been shown to be required for granule exocytosis in eosinophils [48,49,50]. PIP_2_ serves as the precursor for two critical second messengers, inositol 1,4,5-triphosphosphate (InsP_3_) and diacylglycerol (DAG). InsP_3_ triggers a rapid influx of Ca^2+^ which is a mandatory step for the fusion of eosinophil granules to the cell membrane in exocytosis [45]. DAG acts as an activator of protein kinase C (PKC, *PRKC*), an essential downstream mediator of eosinophil exocytosis. An additional phosphoinositide, phosphatidylinositol 3,4,5-triphosphate (PIP_3_), is generated by the action of class I phosphoinositide 3-kinase-δ and γ (PI3K, *PIK3CD* and *PIK3CG*) following CR1 (also known as CD35, *CR1*) receptor stimulation, leading to degranulation responses in eosinophils [48]. The precise intracellular sites of PIP_2_ and PIP_3_ formation in eosinophils are not yet identified but are likely to be expressed at both the plasma and granule membranes. Regions of PIP_2_ and PIP_3_ enrichment in the membrane serve as essential binding sites to anchor numerous intracellular signaling molecules, specifically those with pleckstrin homology domains such as PI-PLC.

Other phospholipases also play a role in eosinophil degranulation responses. Cytosolic phospholipase A2 (cPLA2, *PLA2G4A*) has been shown to be involved in PI-PLC-mediated degranulation responses in eosinophils [50,51]. cPLA2 works in coordination with PI-PLC to play a key role in eosinophil degranulation as well as leukotriene synthesis [50]. As an important enzyme that mediates arachidonic acid release, cPLA2 hydrolyzes nuclear membrane phospholipids at the sn-2 position and generates lysophospholipids and free fatty acids like arachidonic acid, leading to changes in intracellular calcium flux which leads to degranulation [50]. 

Phospholipase D1 (PLD1, *PLD1*) and PLD2 (*PLD2*) may also be involved in eosinophil degranulation responses by generating phosphatidic acid (PA), which accumulates at the membrane near receptors following receptor binding and activation. PA induces negative membrane curvature which is biophysically required to lower the energy barrier for fusion pore formation that facilitates the fusion of granules with the plasma membrane during exocytosis [73]. PLD-derived PA also directly activates PKC, and PA can be converted into DAG to sustain PKC activation [74,75]. However, a direct role for PLD has not yet been demonstrated for eosinophil degranulation. In summary, membrane lipids are an essential component of eosinophil degranulation.

Eosinophils undergo cytolysis, a programmed mechanism of suicidal cell death associated with the extracellular release of intact granules, or degranulation, through adhesion to immobilized immunoglobulins and C3b [43]. In modern reports, this process is frequently referred to as eosinophil extracellular trap cell death or EETosis [17]. This involves the rupture of the eosinophil plasma membrane to release intact cell-free granules and web-like DNA structures that are designed to trap and destroy pathogens. Hence, EETosis contributes to host defence by facilitating the immobilization of pathogens. Both degranulation [48] and EETosis [76] have been shown to be triggered by PI3K activation, suggesting a role for membrane phospholipids in necroptosis, a form of regulated cell death. However, we note that the precise relationship between PI3K activation, cytolysis, EETosis, and other regulated cell death pathways remains incompletely understood. Al-though necroptotic signaling has been suggested as a potential contributor to eosinophil lytic cell death in some experimental settings, its role in eosinophil EETosis has not yet been fully established as discussed below.

### 4.3. Protein Kinases

Protein phosphorylation is an essential event in eosinophil activation that drives exocytosis following receptor stimulation. Phosphorylation is carried out by enzymes known as kinases, which are themselves often phosphorylated by other upstream kinases. This molecular alteration involves the attachment of a phosphate molecule donated by intracellular ATP to a key activation site in the effector protein. The attachment of the phosphate molecule triggers conformational changes that cause the activation of the target protein. Receptor stimulation through the G protein-coupled PAF receptor, for example, leads to phosphorylation of a wide range of kinases which then subsequently activate their respective effector pathways.

Kinases can be differentiated based on their affinity for specific amino acid residues in their target protein. Serine/threonine and tyrosine kinases have been identified as distinct types of kinases involved in receptor signaling. Tyrosine kinases are further differentiated by their integration with the intracellular domain of specific receptors (receptor tyrosine kinases) or as cytosolic enzymes that may be transiently associated with receptors and membranes during cell activation.

Serine/threonine and tyrosine kinases are collectively activated, and often overlap, in eosinophils by a range of receptors expressed in these cells including cytokine (IL-3R, IL-5R, granulocyte/macrophage colony-stimulating factor receptor [GM-CSFR]; *IL3RA*, *IL5RA*, *CSFRA*), chemokine (CCR3, CCR1, CCR4, CCR9, CXCR1-4; names are similar to gene symbols), Siglec 8 (*SIGLEC8*, while in mice this is Siglec F or *Siglecf*), immunoglobulin (principally FcγRIIA [*FCGR2A*] and FcαRI [*FCAR*]), complement (C5aR1, C3aR, CR3 (also an integrin, Mac-1), CR1, CR4, C1qR; *C5AR1*, *C3AR1*, *CR1*, *ITGAM*, *ITGAX*, *CD93*, respectively), Toll-like receptors (TLR2-5, TLR7-9; gene symbols are similar), and integrin (VLA-4, VLA-1, VLA-6, LFA-1; *ITGA4*, *ITGA1*, *ITGA6*, *ITGAL*, respectively) receptors [41]. Many serine/threonine kinases act to prime eosinophils to sustain them and render them hyper-responsive to subsequent triggers, rather than fully initiating degranulation responses.

Eosinophils rely on a series of serine/threonine kinases such as PKC and mitogen-activated kinases (MAPKs) to trigger degranulation responses following receptor stimulation [53,56]. While PKC is expressed as multiple different isoforms, eosinophil degranulation appears to be dependent on the atypical isoform PKCζ (*PRKCZ*) [52]. Blocking overall PKC activity effectively halts both superoxide generation and degranulation in PAF-stimulated human eosinophils, suggesting that specific isoforms are required for these effector functions [55].

Other serine/threonine kinases involved in degranulation are extracellular signal-related kinase (ERK)1 (*MAPK3*)/2 (*MAPK1*) and p38 MAPK (*MAPK14*), which are rapidly activated downstream of chemokine receptors like CCR3 (bound by CCL11, for example) [56]. ERK2, along with p38 MAP kinase, is involved in piecemeal degranulation in response to chemokine stimulation as determined by pharmacological inhibition of these kinases [56]. In mouse eosinophils, p38 MAPK is required for eosinophil migration and degranulation, like their human counterparts, while a role for ERK was not indicated, showing a distinct species-specific pathway for degranulation responses [77].

More recently, a role for a proline-directed serine/threonine kinase, cyclin-dependent kinase 5 (Cdk5, *CDK5*) and its effector molecules, p35 (*CDK5R1*) and p39 (*CDKR2*), has been indicated in eosinophil exocytosis [57]. The regulation of p35 and p39 is controlled by the activation of the ERK/MAPK and Egr-1 (*EGR1*) transcription factor pathways to rapidly synthesize these proteins and attach lipid anchors to them so they reach the membrane before they bind to and activate Cdk5 [78]. At the inner leaflet of the cell membrane, Cdk5 plays a central role in phosphorylating Munc18-3 (*STXBP3*), a regulator of SNARE binding. This results in Munc18c release from syntaxin-4 (*STX4*), allowing SNARE binding and vesicle fusion for subsequent eosinophil degranulation [57].

In cytolysis, eosinophil activation by immunoglobulins and C3b leads to p38 MAP kinase phosphorylation, which then works together with PI3K to activate NADPH oxidase, a superoxide-generating enzyme complex that assembles at the plasma membrane [43,76]. This initiates the respiratory burst, and the reactive oxygen species generated during this process are required for EETosis, a lytic form of eosinophil death. Here, we see that this respiratory burst is a critical event in cytolysis.

Recent studies suggest that the cell’s necroptosis mechanisms are activated involving receptor-interacting protein kinase 3 (RIPK3, *RIPK3*) [43] and mixed lineage kinase domain-like pseudokinase (MLKL, *MLKL*). Specifically, activated RIPK3 targets its primary effector, MLKL, and it is the oligomerization of phosphorylated MLKL that leads to protein clumping at the cell membrane and form pores, which then disrupts the cell, causing cytoplasmic vacuolization, breakdown of the nuclear membrane, and eventually rupture of the cell leading to intact granule release [43]. This is unique from many other cells undergoing necroptosis that usually utilize death receptors like TNF receptors.

However, we note that eosinophil EETosis is not considered synonymous with classical necroptosis [17,18,79,80,81]. Instead, our current understanding is that EETosis shares several mechanistic features with necroptosis, including RIPK3- and MLKL-dependent plasma membrane permeabilization [43], while also exhibiting distinct characteristics such as dependence on NADPH oxidase-derived reactive oxygen species, extracellular chromatin release, and the liberation of intact granules [42]. Here, it is important to note that cytolysis, EETosis, and necroptosis are related but distinct concepts. Specifically, cytolysis describes the morphological outcome of cell rupture, EETosis refers to the eosinophil-specific lytic cell death program associated with extracellular trap formation, and necroptosis denotes a regulated signaling pathway that may contribute to the execution of EETosis under certain conditions. The exact mechanistic relationship between these processes remains yet to be elucidated.

Tyrosine kinases are predicted to induce eosinophil degranulation and respond downstream of immunoglobulin receptors like FcγRIIA or FcαRI [82]. However, evidence for specific tyrosine kinases in eosinophil degranulation responses has not been reported as only broad tyrosine kinase inhibitors have been shown to block degranulation responses in eosinophils [82]. Syk kinase (*SYK*) is a nonreceptor tyrosine kinase that associates directly with immunoglobulin receptors upon activation and can promote respiratory burst responses in eosinophils, although somewhat surprisingly its role in degranulation is not ascertained, even though it has been established as a driver of degranulation in other allergic cells such as mast cells and basophils [83]. Lyn kinase (*LYN*), a member of the *src* family of tyrosine kinases and the non-receptor tyrosine kinase, Jak2 (*JAK2*), are expressed in eosinophils, but these cells do not show the same dependency on these kinases for exocytosis of granule proteins as neutrophils [58], suggesting differentiation in degranulation signaling from other myeloid cells. Lyn and Jak2 kinases are involved in IL-5-stimulated differentiation of eosinophils and their survival, but do not appear to have a role in degranulation responses [58,84]. Instead, the serine/threonine kinase Raf-1 (*RAF1*) is required for exocytosis of granule proteins in response to IL-5R stimulation [58].

Other members of the *src* family of kinases such as Hck (*HCK*) and c-Fgr (*FGR*) are predicted to interact with CCR3 in eosinophils, but the mechanisms underlying a potential role for these kinases in eosinophil degranulation has not yet been identified, although CCL11-induced chemotaxis and membrane ruffling has been shown to be blocked by tyrosine kinase inhibitors [85]. It is postulated that CCR3 activates Hck and c-Fgr through a signaling complex of activated CCR3 bound to cytosolic phosphoproteins that regulate GPCR activity known as β-arrestins (*ARRB1*, *ARRB2*), leading to granule trafficking and exocytosis in eosinophils, although this is not yet established [86].

Among calcium-dependent kinases are PKC which is essential for inducing receptor-mediated degranulation from eosinophils, as well as calmodulin kinase, which phosphorylates the calcium sensor calmodulin that is required for exocytosis in many cell types, while its function in eosinophils is yet to be determined [87]. These proteins are the likely sensors for Ca^2+^ that are activated in permeabilization and patch clamp experiments described above, along with some SNAREs that are also dependent on Ca^2+^ for their activities. In summary, these findings indicate that serine/threonine kinases, ERK1/2, and p38 MAP kinase play a role in regulating the release of granules in response to cytokine and chemokine receptor stimulation in eosinophils, and likely act at an early step proximal to the receptor in this process (Figure 1).

### 4.4. Ras-Related Guanosine Triphosphatases

Exocytosis requires the binding of GTP to numerous intracellular effector molecules, based on the observation that the addition of the nonhydrolyzable analog GTPγS to permeabilized or patch-clamped eosinophils leads to secretion of granule-derived mediators [45,46,71]. This implicates a central role for GTP-binding proteins, specifically guanosine triphosphatases (GTPases), in granule translocation and exocytosis. To date, over 150 well-characterized small monomeric GTPases and 16 distinct genes for α subunits of large heterotrimeric G proteins have been identified in the human genome [88]. While heterotrimeric G proteins typically relocate to the plasma membrane during receptor ligand binding to transduce signals to the cytoplasm, the superfamily of small monomeric Ras GTPases can reside more broadly throughout the cell including the cytoplasm, actin cytoskeleton, or various membrane sites. The wide distribution of Ras GTPases allows these proteins to fulfil a regulatory role in cell activation. Based on their ability to bind to GTP (active state) or GDP (inactive state), Ras GTPases are important switches in cells to turn on or off a signaling event. When bound to high energy GTP, these proteins cleave a phosphate molecule off to form GDP to activate the next effector molecule in signaling. Binding to GTP often induces the association of many cytoplasmic GTPases to membranes or cytoskeletal structures within the cell.

Ras GTPases are further divided into several subfamilies based on their amino acid homology. The largest subfamily is Rab with over 60 members, followed by the Ras subfamily containing 36 members. Other family members are the Arf1 and Sar1 family containing approximately 30 members, the Rho family with about 20 members, and one member for Ran GTPase. Rab, Arf1, and Rho family members are intrinsically involved in transport of proteins through the canonical endoplasmic reticulum-Golgi membrane trafficking and granule exocytosis, with varying roles depending on their locations within the cell. Most Ras GTPases are controlled by two regulatory enzymes, a guanine nucleotide exchange factor (GEF), which activates the GTPase, and a GTPase-activating protein (GAP), which inactivates the protein [88].

Among the Rab subfamily, there are several Rabs that are essential for exocytotic responses in cells. Early studies examining the role of Rabs in eosinophil degranulation responses showed that isoforms of Rab3 (a, b, c, d; *RAB3A*, *RAB3B*, *RAB3C*, *RAB3D*), a putative GTP-binding regulator of exocytotic fusion, were undetectable in eosinophils, at least from guinea pigs [89]. However, later studies in human eosinophils show that transcripts for Rab3 (a, c, and d) were detected [90]. Other Rabs have been implicated in exocytosis such as Rab27A (*RAB27A*), B, Rab10 (*RAB10*), Rab11A/B (*RAB11A/B*), Rab35 (*RAB35*), and Rab37 (*RAB37*) [89].

Of these, only Rab27A GTPase has been shown to serve a role in granule exocytosis from eosinophils so far. Rab27A is a membrane-associated GTPase found in association with crystalloid granules in human and mouse eosinophils [59]. Gene knockout of Rab27A in mouse studies using spontaneously mutated *Ashen* mice were utilized to understand the role of Rab27A in eosinophil degranulation responses. *Ashen*-derived eosinophils exhibited diminished degranulation responses in vitro in response to PAF or calcium ionophore, as determined by EPX release [59].

To understand the in vivo responses of *Ashen* eosinophils, *hE2/IL5* transgenic mice were utilized as a model that is highly stimulatory for eosinophil degranulation, with human eotaxin-2 (CCL24) and IL-5 expressed at elevated levels within the lungs leading to widespread eosinophil degranulation [91]. Eosinophils from *Ashen* mice showed a substantial loss of degranulation responses when intratracheally transferred to double transgenic *hE2/IL5* EPX^−/−^ mice, suggesting that Rab27A is responsible for a substantial majority of the degranulation response in eosinophils. This observation also indicates that receptor-mediated exocytosis through Rab27A is a dominant response in the lungs of *hE2/IL5* mice, rather than cytolysis.

Furthermore, *Ashen* mice exhibit significantly diminished levels of EPX in their airways as well as reduced airway hyperresponsiveness to methacholine following allergic sensitization and challenge with ovalbumin [59]. These observations suggest that Rab27A-dependent eosinophil degranulation responses are essential for promotion of airway hyperresponsiveness, although the *Ashen* mutation is global and it was not possible to confirm this effect arising specifically from eosinophils. Future experiments looking at Rab27A deficiency specifically in eosinophils would be of benefit to determine the precise role of eosinophil degranulation in allergic airway inflammation.

Findings in mouse eosinophils have been confirmed in their human counterparts with the use of nexinhib, a specific inhibitor of Rab27A [92]. Nexinhib significantly inhibited the release of MBP, EPX, and ECP in response to phorbol myristate acetate (PMA) and ionomycin, activators of PKC and Ca ^2+^ increases [92]. These observations indicate that Rab27A expression is crucial for the direction of eosinophils in causing immune regulation or inflammation.

Several downstream effectors are activated by Rab27A including Sec/Munc proteins, such as Munc18. Munc18 has been shown to be expressed in eosinophils in a complex with Cdk5, ostensibly to promote docking and priming of granules for membrane fusion [57].

A second subfamily of Ras GTPases, the Rho subfamily, is also of importance in exocytosis as it serves a role in regulating actin cytoskeletal rearrangement. Remodeling of the actin cytoskeleton is critical for a diverse range of cellular activities, including cell motility (chemotaxis) and exocytosis. The three prototypical members of the Rho subfamily are Rho, Rac, and Cdc42 (*RHOA-D*, *RAC1-3*, *CDC42*) [93,94,95]. Rac is present as three different isoforms, Rac1, Rac2, and Rac3. Rac3 possesses limited tissue distribution and has not been characterized in eosinophils. Rac1 and Rac2 possess 92% homology in their amino acid sequences, with differences concentrated in the final 10 amino acids of their carboxyl termini. It is because of this high homology that both Rac1 and Rac2 serve functionally interchangeable roles in actin cytoskeletal remodeling. Both isoforms are expressed at equivalent levels in eosinophils in mice [47] and in humans, Rac2 was found to be expressed at a greater level than Rac1 [90]. The function of Rac2 in eosinophils has been determined by examining degranulation responses in eosinophils obtained from *Rac2*^−/−^ mice. Gene knockout of Rac2 resulted in 62% diminished degranulation responses measured as EPX release in response to eotaxin-2 or PAF from purified eosinophils from *Rac2^−/−^* mice crossed with *CD2-IL5* transgenic mice (to boost eosinophil yields for experimental work). Responses were not fully inhibited in *Rac2*^−/−^ eosinophils likely because of redundancy with Rac1 expression, which can support Rac2-dependent mechanisms. *Rac2^−/−^* eosinophils are predicted to exhibit a failure in translocation of their crystalloid granules to the cell membrane during receptor stimulation. Thus, the defect in granule exocytosis in these cells is likely to lie within the translocation machinery required to move granules to the cell membrane for docking and fusion. Indeed, Rac2 was shown to be required for the formation of F-actin in response to eotaxin-2 and PAF stimulation [47]. Identification of downstream effector molecules of Rac2 that are responsible for actin cytoskeletal remodeling and/or microtubule rearrangements for granule translocation will be essential for identifying pathways associated with Rac2-mediated granule release (Figure 1).

### 4.5. SNAREs

The final step of exocytosis where the granule membrane fuses with the plasma membrane involves the mutual recognition of granules and target membranes, which is driven by a set of intracellular receptors that guide the docking and fusion of granules. The paradigm for SNARE (SNAP receptor) binding states that secretory vesicles express membrane-bound receptor molecules allowing their specific binding to another set of membrane-bound cognate receptors in target membranes [96].

A highly conserved family of components of a fusion complex of membrane-bound proteins proposed to be essential for vesicular docking and fusion in all cell types was identified decades ago in yeast and neuronal cells, known as SNAREs [96,97,98]. The fusion complex consists of a vesicular SNARE (v-SNARE), recently renamed an R-SNARE based on the position of an arginine residue in the assembled core SNARE complex, which binds to plasma membrane target SNAREs (t-SNAREs), recently renamed as Q-SNAREs for their expression of a glutamine residue in the SNARE complex. The prototypical members of this complex are vesicle-associated membrane protein (VAMP-1, also known as synaptobrevin-1; *VAMP1*) and target membrane-associated syntaxin-1 (*STX1A* and *STX1B* for syntaxin 1A and 1B, respectively) and synaptosome-associated protein of 25 kDa (SNAP-25, *SNAP25*). The fusion of membranes involves the disassembly of the SNARE complex which is proposed to depend on cytosolic NSF (*N*-ethylmaleimide-sensitive factor; *NSF*) and α, β, or γ-SNAP (soluble NSF-attachment protein; *NAPA*, *NAPB*, *NAPG*).

During SNARE binding, the R- and Q-SNAREs form a four-α-helix coiled coil structure that is contributed to by three different molecules with one strand each from VAMP and syntaxin while two strands originate from SNAP-25. The binding region associated with the four α-helices is known as the SNARE motif. The stability of the bonds within the SNARE motif is such that it can withstand detergent treatment even with highly chaotropic detergents like sodium dodecyl sulfate used for sample preparation in immunoblotting [99].

Interestingly, SNARE molecules are exquisitely sensitive to cleavage by clostridial neurotoxins containing zinc endopeptidase activity. These are tetanus toxin (TeNT) and botulinum toxin serotypes (BoNT/A, B, C, D, E, F, and G) [100]. The physical effects of these toxins on human health are the molecular basis of spastic and flaccid paralysis induced by tetanus and botulinum toxin poisoning, respectively. While TeNT and BoNT holotoxins can enter neuronal cells at neuromuscular junctions and spinal ganglia, through their heavy chain components binding to gangliosides on nerves, these molecules are unable to affect non-neuronal tissues as the latter do not express gangliosides [100]. However, other isoforms of SNAREs are expressed in many cells outside of the neuronal system, such as syntaxin-4 and SNAP-23 (*SNAP23*) [101], while VAMP-2 (*VAMP2*) expression overlaps between neuronal and non-neuronal tissues [102]. Other VAMP isoforms such as VAMP-4 (*VAMP4*), VAMP-5 (*VAMP5*), VAMP-7 (*VAMP7*) (formerly known as tetanus toxin-insensitive VAMP or TI-VAMP), and VAMP-8 (*VAMP8*) have been characterized in non-neuronal tissues [103,104,105,106,107,108,109].

Eosinophils have been shown to express VAMP-2, VAMP-7, VAMP-8, syntaxin-4, syntaxin-17 (*STX17*), and SNAP-23 [19,60,61,62,64,65]. VAMP-2 was localized to small secretory vesicles consisting of a population of rapidly mobilizable vesicles transporting the chemokine RANTES (CCL5) to the cell surface for release in response to IFNγ [60]. These findings suggested that VAMP-2 is required for piecemeal degranulation in human eosinophils which is the predominant mechanism of degranulation observed in allergic tissues [36]. The sensitivity of VAMP-2 to cleavage by TeNT was determined by Western blot analysis of purified small secretory vesicles, although it was not possible to test the effects of TeNT on eosinophil degranulation responses as they do not express gangliosides required for binding by the holotoxin [60].

Later studies demonstrated that antibodies to VAMP-2 and VAMP-7 incubated with streptolysin-*O*-permeabilized human eosinophils significantly decreased degranulation responses to Ca^2+^ and GTPγS introduced in cell culture media [110]. Permeabilization of eosinophils with streptolysin-*O* generates large pores (20–30 nm) that easily allow antibodies in the supernatant to penetrate the cell interior and bind to as well as neutralize their target proteins. Exocytosis was measured as supernatant levels of granule proteins in these studies. Using this approach, low concentrations of anti-VAMP-7 antibody impaired the secretion of EPX and EDN while anti-VAMP-8 was without effect [110]. The introduction of anti-VAMP-2 to permeabilized eosinophils modestly and selectively impaired EPX secretion from small vesicles [110]. In summary, eosinophil small secretory vesicles appear to depend on VAMP-2 for secretion while crystalloid granules require VAMP-7 for exocytosis.

In addition to VAMP-2 and VAMP-7, eosinophil granules and small secretory vesicles also express the Q-SNARE syntaxin-17 [65], which were elevated following CCL11 stimulation, a known agonist of piecemeal degranulation. Syntaxin-17 was specifically localized to the secretory granules and vesicles and not endoplasmic reticulum or Golgi complex regions, suggesting a specific role for this SNARE in the secretory pathway for piecemeal degranulation.

We confirmed that VAMP-7 is also expressed in mouse eosinophil crystalloid granules, and that it is required for eosinophil degranulation responses both in vitro and in vivo [63]. Using a floxed VAMP-7 mouse strain that was bred with *eoCRE* mice that express Cre recombinase only in eosinophils [111], we generated offspring that were deficient in VAMP-7 only in eosinophils to determine the impact of this deficiency on eosinophil degranulation and allergic airway inflammation. Following the generation of *VAMP-7*^−/−^*/eoCRE* (*V7/eoCRE*) mice, we crossed these animals with IL-5 transgenic mice to boost eosinophil numbers for experimental work and observed a significant decrease in MBP and EPX secretion in response to PAF, ionomycin, and IL-33 (*IL33*) compared to control eoCRE eosinophils [63]. When *V7/eoCRE* mice were subjected to allergic airway inflammation through ovalbumin sensitization and challenge, we observed a significant reduction in airway hyperresponsiveness to methacholine challenge [63]. No effect was observed on airway inflammation, MBP^+^ eosinophil numbers or other inflammatory cell infiltration, or mucus production in the lungs of *V7/eoCRE* mice following ovalbumin sensitization and challenge. These observations suggest that the major physiological outcome of eosinophil degranulation in allergic airway inflammation is primarily associated with decreased airway resistance as a feature of bronchoconstriction [63]. In summary, SNARE isoforms are likely to play a crucial role in the regulation of granule fusion in eosinophils.

Although SNARE proteins syntaxin-4 and SNAP-23 play a role in eosinophil granule exocytosis, they may not have an impact on mitochondrial or nuclear DNA release associated with extracellular trap formation as determined in activated neutrophils [112]. This suggests that EETosis may be a mechanism that does not require specific SNARE-mediated membrane fusion events.

### 4.6. Other Regulatory Molecules of Exocytosis in Eosinophils

Chaperone proteins known as Sec1/Munc18-like (SM) proteins have been implicated in regulation of eosinophil exocytosis. Eosinophils express Munc18c (syntaxin-binding protein 3; *STXBP3*), which binds tightly to syntaxin-4, maintaining it in a closed, inactive configuration to prevent premature degranulation. Upon eosinophil activation, Cdk5 phosphorylates Munc18c, which then releases syntaxin-4, allowing the core fusion machinery to assemble [57].

In the process of autophagy, a highly regulated catabolic process where cellular contents are enveloped in double-membraned vesicles called autophagosomes, followed by their degradation by proteolytic lysosomal enzymes, numerous proteins are involved in the autophagic pathway including autophagy-related protein 5 (ATG5; *ATG5*) which is expressed in eosinophils [66]. Eosinophils lacking ATG5 exhibit reduced differentiation, as well as, somewhat surprisingly, elevated degranulation responses [113]. These observations suggest that ATG5 normally functions to suppress degranulation responses through reduced MAPK activity, with reduced phosphorylation of Stat3 (*STAT3*), p38, and ERK1/2 kinases observed in *Atg5*-knockout eosinophils following GM-CSF priming [113]. Thus, autophagy is a process that has significant impact on eosinophil degranulation responses.

Numerous other proteins have been implicated in SNARE-mediated exocytosis in a range of other cell types, including synaptotagmins, complexins, and cytoskeletal remodeling proteins including actin-severing proteins (cofilin and gelsolin) as well as annexins [114,115]. These proteins are postulated to also have a role in eosinophil degranulation, although experimental evidence is yet to be reported for their involvement.

## 5. Summary

These experimental observations reveal a large group of intracellular signaling molecules that regulate the translocation of granules to the cell membrane for their docking and fusion to release the contents of these organelles to the exterior of cells. Several of these molecules are natural targets for bacterial toxins, highlighting their central role in regulating bactericidal mediator release. It may be possible to utilize specific inhibitors or exploit the use of bacterial toxins that target these exocytotic regulatory factors as tools to prevent or modulate tissue-damaging eosinophil degranulation. Eosinophil degranulation is an important event in inflammatory diseases such as allergic asthma, allergic rhinitis, and chronic rhinosinusitis. Products of eosinophil degranulation, including MBP and EPX which is specific to eosinophils, have been shown to increase in proportion to severity of disease in the airways of patients with these diseases [116,117]. Elevated EPX in sputum has been associated with severe airflow obstruction, exacerbations, and mucus plugging in asthma [118]. Further analysis of the signaling pathways that specifically activate the release of granules and vesicles for the trafficking of proinflammatory proteins and molecules in eosinophils may guide the development of novel drugs that will prevent eosinophil degranulation in airway diseases and related inflammatory disorders.

## Figures and Tables

**Table 1:** Intracellular regulatory factors involved in eosinophil exocytotic responses.

Regulatory Factor	Site Within Cell	References
**Phospholipids and related molecules**		
PIP_2_	Inner leaflet of plasma membrane	[48,49]
PIP_3_	Inner leaflet of plasma membrane	[48]
PI-PLC	Cytoplasm	[48,49,50]
InsP_3_	Cytoplasm	[45]
DAG	Cytoplasm	[45]
PLA2	Cytoplasm	[50,51]
**Kinases (serine/threonine)**		
PKC	Cytoplasm	[52,53,54,55]
p38 MAPK	Cytoplasm	[56]
ERK2	Cytoplasm	[56]
Cdk5	Cytoplasm	[57]
Raf-1	Cytoplasm	[58]
**Small monomeric GTPases**		
Rab27A	Granule membrane	[59]
Rac1/Rac2	Cytoplasm	[47]
**SNAREs**		
VAMP-2	Small secretory vesicles	[60,61]
VAMP-7	Crystalloid granules, small secretory vesicles	[62,63]
VAMP-8	Crystalloid granules, small secretory vesicles	[62]
Syntaxin-4	Inner leaflet of plasma membrane	[64]
Syntaxin-17	Granule membrane	[65]
SNAP-23	Inner leaflet of plasma membrane	[64]
**Additional proteins**		
Munc18c	Cytoplasm	[57]
ATG5	Cytoplasm	[66]

## Data Availability

No new data were created or analyzed in this study.
